# CH_3_NH_3_PbBr_3_ Perovskite Single-Crystal X-Ray Photon-Counting Detection Based on Multi-Layer Electrodes

**DOI:** 10.3390/s26103030

**Published:** 2026-05-11

**Authors:** Songchao Wang, Hanwen Zhang, Gangyi Chen, Yuzhu Pan, Yulian Zhang, Qianqian Huang, Jinbao Chen, Xin Wang

**Affiliations:** 1 National Key Laboratory of Aerospace Mechanism, Nanjing University of Aeronautics and Astronautics, Nanjing 210016, China; wsc45137210@126.com (S.W.);; 2 Shanghai Institute of Spacecraft Equipment, Shanghai 200240, China; 3 Shanghai Aerospace Equipments Manufacturer Co., Ltd., Shanghai 200245, China; kuangzhanshi_xp@hotmail.com; 4 Key Laboratory of Semiconductor Display Materials and Chips, Suzhou Institute of Nano-Tech and Nano-Bionics, Chinese Academy of Sciences, Suzhou 215123, China; yzpan2024@sinano.ac.cn; 5 College of Physics, Nanjing University of Aeronautics and Astronautics, Nanjing 210016, China; 6 School of Information Technology, Jiangsu Open University, Nanjing 210017, China; huangqq@jsou.edu.cn

**Keywords:** CH_3_NH_3_PbBr_3_ single crystal, multi-layer electrode, X-ray photon counting detection

## Abstract

**Highlights:**

**What are the main findings?**

- Au/Pt/Ti multi-layer is a good choice for electrodes for CH_3_NH_3_PbBr_3_ perovskite single crystals.
- Low-noise CH_3_NH_3_PbBr_3_ perovskite single crystals could be applied in X-ray photon-counting detection and imaging.

**What are the implications of the main findings?**

- Scientists and engineers could try multi-layer electrodes for CH_3_NH_3_PbBr_3_ perovskite single crystals.
- More lead halide perovskite single crystals should be considered in X-ray photon counting detectors.

**Abstract:**

CH_3_NH_3_PbBr_3_ (MAPbBr_3_) single crystals have shown great potential in X/γ-ray detection. However, stable electrodes for MAPbBr_3_ single crystals still remain challenging. In this work, multi-layer electrodes including Au, Au/Ti and Au/Pt/Ti are investigated. Through I-V characterization, Au/Pt/Ti shows Ohmic contact behavior and the lowest dark current. The potential contact is also confirmed by the Kelvin force probe. Based on these low-noise electrodes, 59.5 keV monochromatic X-ray photon-counting detection and imaging is demonstrated. This work provides useful information for electrode design in lead halide perovskite-based optoelectronic devices.

## 1. Introduction

X-ray detection technologies play a critical role in a wide range of applications, including medical imaging, industrial non-destructive testing, and security checks [1,2,3]. Among various detection modalities, photon-counting detection has emerged as a next-generation paradigm due to its superior capabilities in energy resolution, contrast-to-noise ratio, and reduced radiation dose compared to conventional energy-integrating systems [4,5,6,7,8,9]. The success of photon-counting detection, however, heavily relies on the availability of high-performance semiconductor materials that exhibit both efficient X-ray absorption and excellent charge transport properties [10].

In recent years, lead halide perovskites have garnered significant attention as promising candidates for X-ray detection, owing to their exceptional optoelectronic properties, including high atomic numbers, large mobility-lifetime (μτ) products, and low-cost solution-processability [11,12,13,14]. Among kinds of lead halide perovskites, methylammonium lead bromide (MAPbBr_3_, MA=CH_3_NH_3_) stands out as a particularly attractive material for X-ray photon counting applications, as it combines strong X-ray attenuation capability with high resistivity and quick growth speed from solution precursor [15,16,17]. These attributes have enabled MAPbBr_3_ single-crystal-based X-ray detectors to achieve impressive sensitivity and the lowest detection limits, positioning them as viable alternatives to conventional materials such as amorphous selenium, cadmium zinc telluride, and silicon [18,19,20,21]. In previous work, a CsPbBr3 single crystal was used as a high flux X-ray photon counting detector with a count rate of 263 kcps [9]. Further, the MAPbI_3_ single crystal was applied in X-ray photon counting detectors with the extremely low noise-equivalent dose of 90 pGy_air_ [5].

Despite these advances, the application of MAPbBr_3_ single crystals in X-ray photon-counting detection remains challenging, primarily due to issues related to device stability, high dark current, and charge trapping under high electric fields [22,23,24]. The electrode configuration plays a crucial role in governing charge collection efficiency and suppressing dark current in perovskite X-ray detectors. Conventional single-layer metal electrodes such as Au, Ag, Ti and Ga often suffer from inefficient charge extraction, interfacial reactions with the MAPbBr_3_ single crystals, and increased leakage currents, limiting the detector’s ability to operate stably under sustained high bias and X-ray irradiation [25,26,27,28]. While previous studies have largely focused on optimizing the active layer composition and interface engineering, relatively little attention has been paid to the rational design of multi-layer electrode architectures tailored for photon counting mode, particularly in the context of MAPbBr_3_ single crystal-based devices [5,29].

In previous reports, multi-layer electrodes including MoOx/Cu/Ag/MoOx, IZrO/IZO, AZO/Au/AZO, AZO/Cu/Ag/AZO and MoOX/Cr/Al were used as low-resistivity and high-transmittance electrodes applied in solar cell [30,31,32,33,34]. However, these electrodes are not stable under high bias when in contact with the MAPbBr_3_ single crystal.

To address these limitations, we propose the implementation of multi-layer electrode structures employing Au/MAPbBr_3_/Au, Au/Ti/MAPbBr_3_/Ti/Au and Au/Pt/TiMAPbBr_3_/Ti/Pt/Au configurations. These multi-layer architectures are designed to combine the advantages of each metal layer: Ti serves as an adhesion layer, where Ti-N bonds are formed at the interface of MAPbBr_3_/Ti [35]; Pt functions as an isolating layer to mitigate interfacial reactions and ion migration; while Au ensures high conductivity. By systematically comparing the performance of MAPbBr_3_ X-ray detectors with single-layer and multi-layer electrodes, we investigate the influence of electrode architecture on 59.5 keV photon-counting detection and imaging. In this work, low-activity ^241^Am radioactive gamma-ray sources were used as a low-dose monochromatic X-ray source.

Our results demonstrated that the multi-layer electrode strategy, particularly the Au/Pt/Ti/MAPbBr_3_/Ti/Pt/Au configuration, significantly enhances both the detection performance and long-term stability of MAPbBr_3_-based photon counting X-ray detectors. The improved charge extraction, suppressed dark current, and mitigated interfacial degradation achieved through this rational electrode design offer a promising pathway toward practical and reliable perovskite-based photon-counting X-ray detection systems.

## 2. Materials and Methods

Materials: Lead bromide (PbBr_2_; 99%) and methylammonium bromide (MABr, 99%) were purchased from Sigma Aldrich, St. Louis, MO, USA. Dimethyl sulfoxide (DMSO, 99.9%) and dimethylformamide (DMF, 99.9%) were obtained from Aladdin. Metallic gold was purchased from Chinese reagent, China. All the commercial products were used as received.

Growth of MAPbBr_3_ perovskite single crystals. High-quality MAPbBr_3_ perovskite single crystals were grown by inverse temperature crystallization [36,37,38]. Specifically, 1 M MABr and 1 M PbBr_2_ were dissolved in DMF solution. The solutions were filtered through a PTFE filter with a 0.22 μm pore size. The filtrate was then transferred to a culture dish, which was placed on a programmable heating station (IKA-RET control-visc), and the growth temperature was 65 to 85 °C with a rate of 1 °C h^−1^. For the metal electrodes, electrodes were deposited on the face of MAPbBr_3_ perovskite single crystals by a metal mask under a vacuum of 6 × 10^−4^ Pa.

Characterization of the MAPbBr_3_ perovskite single crystals. X-ray diffraction (XRD) patterns were obtained using an X’TRA system with a Cu target (Thermo Fisher Scientific, Ecublens, Switzerland). Optical absorption spectra were measured by UV-vis spectroscopy range from 300 to 2000 nm (Lab Tech Bluestar, Ortenberg, Germany). The ultraviolet photoelectron spectroscopy (UPS) was obtained using a PHI 5000 VersaProbe (ULVAC-PHI, Chigasaki, Japan). The PL and PL decay processes were measured using a SpectraMax instrument (Molecular Devices, San Jose, CA, USA). The Kelvin force probe microscopy was used with a Dimension Icon (Bruker, Herzogenrath, Germany). For the response time measurement, ^241^Am was used as the radioactive source, a high voltage bias (0–50V) was applied to the device, and the signal was input into a pre-amplifier 142PC (ORTEC, Oak Ridge, TN, USA). The response to each alpha particle was traced using a Keysight oscilloscope DMOX4054A (Keysight Technologies, Santa Rosa, CA USA). Characterization of current–voltage was measured using a Keithley 4200SC semiconductor analyzer (Keithley, Solon, OH, USA).

Set-up of the X-ray photon-counting read-out circuits. The pre-amplifier 142PC (ORTEC, Oak Ridge, TN, USA). The shaping amplifier and baseline restorer (CREMAT, West Newton, MA, USA). The high-voltage and low-voltage DC sources (DONGWEN HIGH VOLTAGE, Tianjing, China). And the multi-channel analyzer (AP Techno, Hachimantai, Japan).

## 3. Results

**Figure 1:**
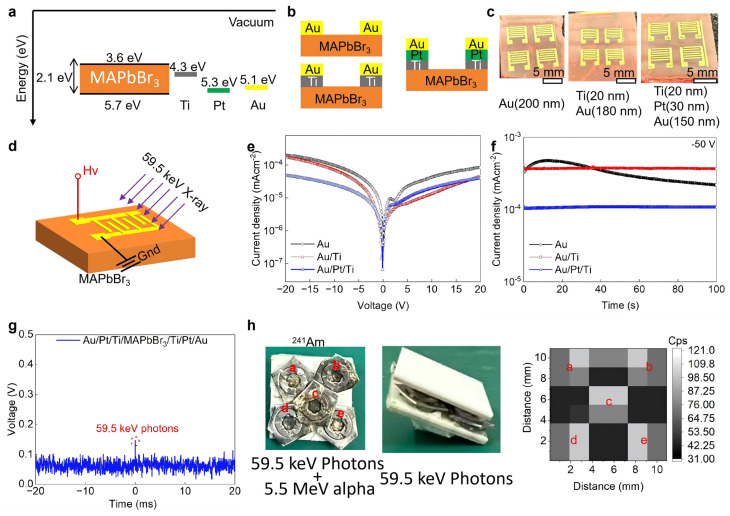
(**a**) Energy band diagram of MAPbBr_3_ single crystal and different metals. (**b**) Device structure. (**c**) Optical photo of devices with different electrodes. (**d**) Diagram of the device under bias and 59.5 keV radiation. (**e**) I-V characterization of MAPbBr_3_ single crystals with different electrodes. (**f**) I-T characterization of MAPbBr_3_ single crystals with different electrodes under −50 V. (**g**) 59.5 keV photon response of Au/Pt/Ti/MAPbBr_3_/Ti/Pt/Au. (**h**) Image of five ^241^Am source, (**a**–**e**) represent the five different locations of ^241^AM source.

As shown in Figure 1a, the energy band of the MAPbBr_3_ single crystal was investigated. The band gap was fitted to 2.1 eV by the Talc plot (Figure S1). And the valence band maximum was measured at 5.7 eV by UPS (Figure S2). And the work function of Au, Pt, and Ti were chosen to be 5.1 eV, 5.3 eV and 4.3 eV from published work, respectively. Figure 1b shows the structure of detectors made of MAPbBr_3_ single crystals with different electrodes, including Au/MAPbBr_3_/Au, Au/Ti/MAPbBr_3_/Ti/Au and Au/Pt/Ti/MAPbBr_3_/Ti/Pt/Au. An interdigitated electrode was chosen as shown in Figure 1c; the thickness of the three types of electrodes was 200 nm, and the area of the active area was 25 mm^2^. Here, ^241^Am radioactive sources, which emit 59.5 keV photons, were used as a monochromatic X-ray source. As shown in Figure 1d, the 59.5 keV monochromatic X-ray photons would incident on the detectors, while high voltage bias and ground would connect to each electrode, respectively.

Figure 1e shows the current density–voltage characterization of detectors with different electrode structures. The Au/Pt/Ti multi-layer electrodes show nearly linear behavior, while the Au electrode or Au/Ti electrode shows back-to-back Schottky behavior. And the I-V characterization with different temperatures is shown in Figure S3. The Au/Pt/Ti multi-layer seems to form a more Ohmic contact with the MAPbBr_3_ single crystal. Further, the dark current density under −50 V bias was investigated in Figure 1f; the Au/Pt/Ti multi-layer device shows the lowest dark current of 107 nA cm^−2^, which was much lower than the Au/Ti device and the Au device of 372 nA cm^−2^ and 227~445 nA cm^−2^, respectively. For MAPbBr_3_ single crystals with only Ti and Pt, the I-V characterization is shown in Figure S4.

For the Ti/MAPbBr_3_ single crystal interface, Wenqing Zhang and co-author have demonstrated that Ti−N bonds formed at the interface of Ti/MAPbBr_3_ single crystal effectively inhibit the electrochemical reaction and ultimately improve the operating stability under a high electric field [35]. However, the Ti electrode was easily oxidized in air. In addition, a Ti/Au electrode would cause significant inter-layer diffusion between Au and Ti, which could lead to device degradation. Thus, inserting a 20 nm thickness Pt layer could effectively prevent Ti segregation [39].

Owing to the low and stable dark current, the Au/Pt/Ti multi-layer device was used to detect 59.5 keV photons. As shown in Figure 1g, a clear potential pulse induced by a 59.5 keV photon can be observed. In contrast, for the Au/Ti multi-layer device and the Au-only device, the noise levels were too high to discriminate the useful signal (Figure S5). The response to alpha particles is presented in Figure S6.

Furthermore, five ^241^Am radioactive sources were used as five point-like X-ray sources. A 1 mm thick Al_2_O_3_ ceramic sheet was placed to filter out alpha particles, allowing only 59.5 keV photons to pass through, as illustrated in Figure 1h. By analyzing the count rate (counts per second) of the Au/Pt/Ti multi-layer device at different positions, we successfully obtained an X-ray photon counting image.

The five ^241^Am radioactive sources emit only approximately 1000 59.5 keV photons per second, resulting in a photon injection rate of just 100 cm^−2^ s^−1^. According to the formula $D_{\mathrm{air}}=\varphi \times (\frac{\mu_{\mathrm{en}}}{\rho })\times E$, where D_air_ is the dose rate, φ is the photon injection rate, E is the energy of each photon and $\frac{\mu_{\mathrm{en}}}{\rho }$ is the mass energy absorption coefficient, which is 0.0303 cm^2^ g^−1^ for air [32], the calculated dose rate is only 0.03 nGy s^−1^. This value is far below the lowest detectable dose rate of typical energy-integrating X-ray detectors.

## 4. Discussion

**Figure 2:**
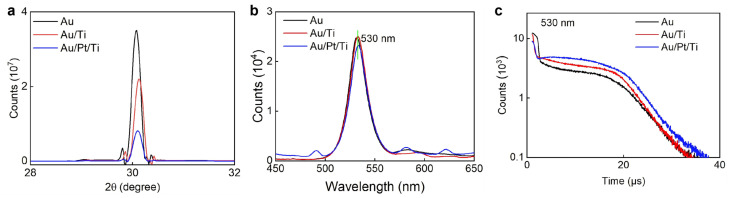
Characterization of MAPbBr_3_ single crystals with different electrodes: (**a**) X-ray diffraction. (**b**) Photoluminescence under 350 nm. (**c**) Time-resolved photoluminescence of 530 nm.

After multi-layer electrode deposition, we further confirmed the surface property of MAPbBr_3_ single crystals. Since the deposition temperature for metal Pt is much higher than that of Au, the high-temperature-vaporized Pt or Au may destroy the surfaces of MAPbBr_3_ single crystals. As shown in Figure 2a, the surface property of MAPbBr_3_ single crystals was first investigated by X-ray diffraction. The full width half height (FWHM) for 30.080 degrees was 0.2, 0.2 and 0.18 degrees for the Au, Au/Ti and Au/Pt/Ti devices, respectively. The X-ray diffraction shows that the crystallization quality of the three MAPbBr_3_ single crystals after electrode deposition was close. Further, the photoluminescence of three MAPbBr_3_ single crystals after electrode deposition was investigated in Figure 2b. The peak of the photoluminescence was located at 530 nm, and the photoluminescence intensity was nearly equal. And the time-resolved photoluminescence of 530 nm was further investigated in Figure 2c. Through exponential decay fitting, the surface lifetime was 0.32 ± 0.02, 0.37 ± 0.02 and 0.35 ± 0.04 μs, and the bulk lifetime was 10.5 ± 3.7, 10.5 ± 2.3 and 14.2 ± 4.5 μs for the Au, Au/Ti and Au/Pt/Ti devices, respectively. As a result, the dark current density and noise behavior were attributed to the different electrodes, not to the crystallization quality differences in MAPbBr_3_ single crystals.

**Figure 3:**
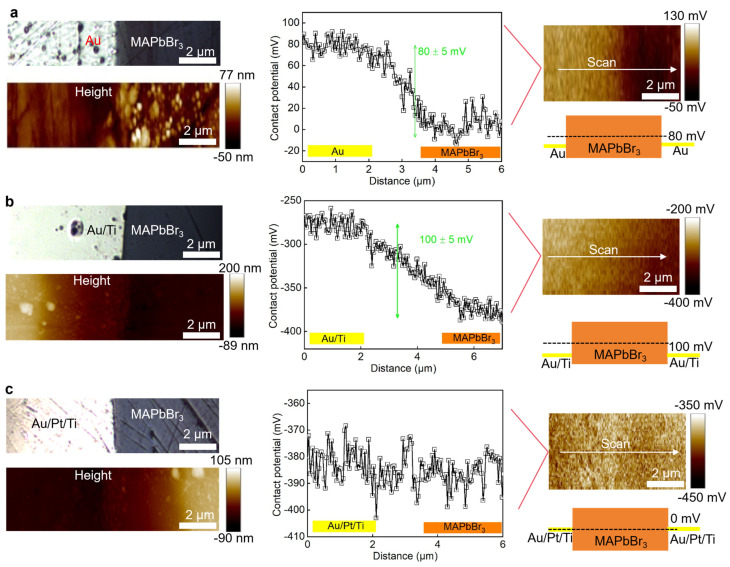
Kelvin force microscopy results: (**a**) Au/MAPbBr_3_. (**b**) Au/Ti/MAPbBr_3_. (**c**) Au/Pt/Ti/MAPbBr_3_.

To further understand the metal–MAPbBr_3_ single crystal contact, here, Kelvin force microscopy (KFM) was used to measure the contact potential of different electrodes. The KFM results for the Au device are shown in Figure 3a; the roughness was 5 ± 1 and 25 ± 3 nm for the Au area and the MAPbBr_3_ single crystal region, respectively. And the contact potential was measured at 0.15 ± 0.02 V, resulting in an energy barrier of 80 mV in the Au-MAPbBr_3_ interface. Similarly, for the Au/Ti device, the roughness was 3 ± 1 and 15 ± 3 nm for the Au/Ti area and MAPbBr_3_ single crystal region, respectively. Further, the contact potential was measured at 0.15 ± 0.02 V, resulting in an energy barrier of 100 mV in the Au/Ti-MAPbBr_3_ interface. For the Au/Pt/Ti device, the roughness was 3 ± 1 and 15 ± 3 nm for the Au/Ti area and MAPbBr_3_ single crystal region, respectively. What’s interesting is that we can not observe a clear potential difference between Au/Pt/Ti and MAPbBr_3_ single crystal. It also explained the Ohmic behavior as shown in the current density–voltage characterization.

**Figure 4:**
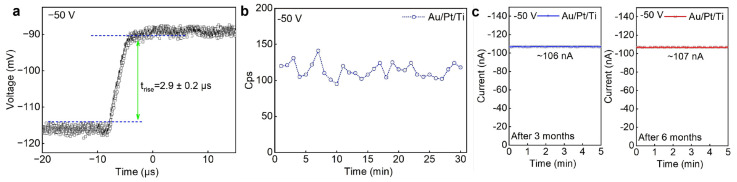
(**a**) Response time to single 59.5 keV photon. (**b**) Count per second stability. (**c**) Long-time stability of Au/PT/Ti/MAPbBr_3_/Ti/Pt/Au.

As shown in Figure 4a, the response speed for the Au/Pt/Ti device, at −50 V bias, the response speed reached 2.9 ± 0.2 μs, corresponding to the maximum count rate of 34.4 k s^−1^. As shown in Figure 4b, the counting stability of the Au/Pt/Ti device was 110 ± 15 for 59.5 keV photons for 30 min, demonstrating not bad counting stability. The Au/Pt/Ti device was also very stable. As shown in Figure 4c, the dark current after 6 months (107 nA) maintained almost the same dark current after 3 months (106 nA) stored in ambient. And the long-term irradiation stability was investigated in Figure S7.

## 5. Conclusions

In this work, multi-layer electrodes including Au, Au/Ti and Au/Pt/Ti were investigated. Au and Au/Ti would form energy barriers when in contact with MAPbBr_3_ single crystals, while Au/Pt/Ti could form Ohmic contact with MAPbBr_3_ single crystals. Au/Pt/Ti multi-layer electrode shows the lowest dark current and noise, which enables 59.5 keV photon-counting detection and imaging. This work provided useful information about the electrode structure for lead halide perovskite-based optoelectronic devices.

## Data Availability

All data supporting this study are included in the article and its Supplementary Information (SI).

## Supplementary Materials

The following supporting information can be downloaded at https://www.mdpi.com/article/10.3390/s26103030/s1. Figure S1: Absorption curve of MAPbBr3 single crystal; Figure S2: UPS result of MAPbBr3 single crystal; Figure S3: Temp-dependent I-V characterization of Au/Pt/Ti device. Figure S4: I-V characterization results of MAPbBr3 single crystal with only Ti and Pt electrodes; Figure S5: Noise of Au, Au/Ti and Au/Pt/Ti under −50 V bias; Figure S6: Alpha response of Au/Pt/Ti device. Figure S7: X-ray irradiation stability.

## References

[1] Liu Z., Wang X., Lei W. (2025). Solution-processed epitaxial growth of PIN photodiodes made of MAPbBr_3_ single crystals for high energy resolution gamma-ray spectroscopy. J. Mater. Chem. C.

[2] Park A., Park B., Seo J., Byun J., Park S.-J., Yoon S.-P., Ko J., Kim J., Lee W., Lee M.-J. (2025). Exploration on high-energy and high-dose rate X-ray detection with Bridgman-grown CsPbBr_3_ single crystal. Nucl. Eng. Technol..

[3] Peng J., Xia C.Q., Xu Y., Li R., Cui L., Clegg J.K., Herz L.M., Johnston M.B., Lin Q. (2021). Crystallization of CsPbBr_3_ single crystals in water for X-ray detection. Nat. Commun..

[4] Li M., Wang S., Wood A., Yeager J.D., Stepanoff S.P., Adler J.C., Shi Z., Wang J., Li Z., Wolfe D.E. (2025). Defect repairing in lead bromide perovskite single crystals with biasing and bromine for X-ray photon-counting detectors. Nat. Mater..

[5] Sakhatskyi K., Turedi B., Matt G.J., Wu E., Sakhatska A., Bartosh V., Lintangpradipto M.N., Naphade R., Shorubalko I., Mohammed O.F. (2023). Stable perovskite single-crystal X-ray imaging detectors with single-photon sensitivity. Nat. Photonics.

[6] Yang Y., Chen X., Jia Z., Liu Y., Lin Q. (2025). Device Engineering of Bismuth-Based Chalcogenides for Low-Noise, Near-Infrared Photon-Counting. ACS Mater. Lett..

[7] Zhou Y., Fei C., Uddin M.A., Zhao L., Ni Z., Huang J. (2023). Self-powered perovskite photon-counting detectors. Nature.

[8] Rodesch P.-A., Richtsmeier D., Guliyev E., Iniewski K., Bazalova-Carter M. (2023). Comparison of Threshold Energy Calibrations of a Photon-Counting Detector and Impact on CT Reconstruction. IEEE Trans. Radiat. Plasma Med. Sci..

[9] Pan L., He Y., Klepov V.V., De Siena M.C., Kanatzidis M.G. (2022). Perovskite CsPbBr_3_ Single Crystal Detector for High Flux X-Ray Photon Counting. IEEE Trans. Med. Imaging.

[10] Iwanczyk J.S., Nygard E., Meirav O., Arenson J., Barber W.C., Hartsough N.E., Malakhov N., Wessel J.C. (2009). Photon Counting Energy Dispersive Detector Arrays for X-ray Imaging. IEEE Trans. Nucl. Sci..

[11] Datta A., Fiala J., Motakef S. (2021). 2D perovskite-based high spatial resolution X-ray detectors. Sci. Rep..

[12] Fratelli I., Basiricò L., Ciavatti A., Margotti L., Cepić S., Chiari M., Fraboni B. (2024). Real-Time Radiation Beam Monitoring by Flexible Perovskite Thin Film Arrays. Adv. Sci..

[13] Wei H., Huang J. (2019). Halide lead perovskites for ionizing radiation detection. Nat. Commun..

[14] Shooshtari M., Kim S.-Y., Pahlavan S., Serrano-Gotarredona T., Bisquert J., Linares-Barranco B. (2026). Bio-Inspired Spike-Timing-Dependent Plasticity Learning with Metal Halide Perovskites: Toward Artificial Synaptic Functionality. ACS Appl. Mater. Interfaces.

[15] Cho Y., Jung H.R., Kim Y.S., Kim Y., Park J., Yoon S., Lee Y., Cheon M., Jeong S.-y., Jo W. (2021). High speed growth of MAPbBr_3_ single crystals via low-temperature inverting solubility: Enhancement of mobility and trap density for photodetector applications. Nanoscale.

[16] Wang K.-H., Li L.-C., Shellaiah M., Wen Sun K. (2017). Structural and Photophysical Properties of Methylammonium Lead Tribromide (MAPbBr_3_) Single Crystals. Sci. Rep..

[17] Wang C., Ecker B.R., Wei H., Huang J., Gao Y. (2018). Environmental Surface Stability of the MAPbBr_3_ Single Crystal. J. Phys. Chem. C.

[18] Eom K., Lee J.T., Oschatz M., Wu F., Kaskel S., Yushin G., Fuller T.F. (2017). A stable lithiated silicon–chalcogen battery via synergetic chemical coupling between silicon and selenium. Nat. Commun..

[19] Song Y., Jackson M., Iniewski K. Recent Results in CZT Detectors for PCCT. Proceedings of the 2023 IEEE Nuclear Science Symposium, Medical Imaging Conference and International Symposium on Room-Temperature Semiconductor Detectors (NSS MIC RTSD).

[20] Zhou B., Jie W., Wang T., Xu Y., Yang F., Yin L., Zhang B., Nan R. (2017). Growth and Characterization of Detector-Grade Cd_0_._9_Zn_0_._1_Te Crystals by the Traveling Heater Method with the Accelerated Crucible Rotation Technique. J. Electron. Mater..

[21] He X., Deng Y., Ouyang D., Zhang N., Wang J., Murthy A.A., Spanopoulos I., Islam S.M., Tu Q., Xing G. (2023). Recent Development of Halide Perovskite Materials and Devices for Ionizing Radiation Detection. Chem. Rev..

[22] Yang Y., Yan Y., Yang M., Choi S., Zhu K., Luther J.M., Beard M.C. (2015). Low surface recombination velocity in solution-grown CH_3_NH_3_PbBr_3_ perovskite single crystal. Nat. Commun..

[23] Cao M., Tian J., Cai Z., Peng L., Yang L., Wei D. (2016). Perovskite heterojunction based on CH_3_NH_3_PbBr_3_ single crystal for high-sensitive self-powered photodetector. Appl. Phys. Lett..

[24] Zhang H., Yu T., Wang C., Jia R., Pirzado A.A.A., Wu D., Zhang X., Zhang X., Jie J. (2022). High-Luminance Microsized CH_3_NH_3_PbBr_3_ Single-Crystal-Based Light-Emitting Diodes via a Facile Liquid-Insulator Bridging Route. ACS Nano.

[25] Shen N., Gao T., Ouyang X., Bayikadi K.S., Duan Z., Xiao B., He X., Wang Y., Qin H., Sun Q. (2024). Enhancing Gamma-Ray Spectral Resolution in Perovskite CsPbBr_3_ Detectors through Dark Current Reduction with Guard Ring Electrodes. ACS Photonics.

[26] Klepov V.V., De Siena M.C., Pandey I.R., Pan L., Bayikadi K.S., Butun S., Chung D.Y., Kanatzidis M.G. (2023). Laser Scribing for Electrode Patterning of Perovskite Spectrometer-Grade CsPbBr_3_ Gamma-ray Detectors. ACS Appl. Mater. Interfaces.

[27] Song S., Yoon A., Jang S., Lynch J., Yang J., Han J., Choe M., Jin Y.H., Chen C.Y., Cheon Y. (2023). Fabrication of p-type 2D single-crystalline transistor arrays with Fermi-level-tuned van der Waals semimetal electrodes. Nat. Commun..

[28] Besleaga C., Abramiuc L.E., Stancu V., Tomulescu A.G., Sima M., Trinca L., Plugaru N., Pintilie L., Nemnes G.A., Iliescu M. (2016). Iodine Migration and Degradation of Perovskite Solar Cells Enhanced by Metallic Electrodes. J. Phys. Chem. Lett..

[29] Shrestha S., Tsai H., Yoho M., Ghosh D., Liu F., Lei Y., Tisdale J., Baldwin J., Xu S., Neukirch A.J. (2020). Role of the Metal–Semiconductor Interface in Halide Perovskite Devices for Radiation Photon Counting. ACS Appl. Mater. Interfaces.

[30] Jiang S., Feng L., Zhang W., Liu H., Liu H., Liu Y., Li B., Wu L., Liu X., Wang X. (2022). Indium-free flexible perovskite solar cells with AZO/Cu/Ag/AZO multilayer transparent electrodes. Sol. Energy Mater. Sol. Cells.

[31] Liu H., Lang R., Jiang S., Lu W., Zhang W., Feng L., Liu H., Wu L., Liu X., Wang X. (2021). Bifacial semitransparent perovskite solar cells with MoOx/Cu/Ag/MoOx multilayer transparent electrode. Sol. Energy.

[32] Han W., Li S., Wang J., Shi B., Huang Q., Zhao Y., Zhang X. (2025). IZrO/IZO multilayer thin film as transparent electrode in perovskite/silicon tandem solar cell. J. Power Sources.

[33] Dang T.-V., Pammi S.V.N., Choi J., Yoon S.-G. (2017). Utilization of AZO/Au/AZO multilayer electrodes instead of FTO for perovskite solar cells. Sol. Energy Mater. Sol. Cells.

[34] Kim W., Kim S., Choi I., Lee S., Yu S., Kim Y.Y., Koo B., Ko M.J. (2026). High-Performance Noble-Metal-Free Perovskite Solar Cells Enabled by MoOX/Cr/Al Multilayer Electrodes. Adv. Energy Mater..

[35] Zhang W., Wang H., Chen Z., Wang P., Liu X., Dong H., Zhao J., Cui Y., Shao Y. (2024). High-Performance and Stable Perovskite X-ray Detection and Imaging Based on a Ti Cathode. ACS Appl. Mater. Interfaces.

[36] Maculan G., Sheikh A.D., Abdelhady A.L., Saidaminov M.I., Haque M.A., Murali B., Alarousu E., Mohammed O.F., Wu T., Bakr O.M. (2015). CH_3_NH_3_PbCl_3_ Single Crystals: Inverse Temperature Crystallization and Visible-Blind UV-Photodetector. J. Phys. Chem. Lett..

[37] Saidaminov M.I., Abdelhady A.L., Murali B., Alarousu E., Burlakov V.M., Peng W., Dursun I., Wang L., He Y., Maculan G. (2015). High-quality bulk hybrid perovskite single crystals within minutes by inverse temperature crystallization. Nat. Commun..

[38] Wang X., Huang Y., Lei W., Li Q., Zhang X., Khan Q., Wang B. (2017). Asymmetrical Photodetection Response of Methylammonium Lead Bromide Perovskite Single Crystal. Cryst. Res. Technol..

[39] Hoshino Y., Saito Y., Nakata J. (2010). Interdiffusion Analysis of Au/Ti and Au/Pt/Ti Electrode Structures Grown on Diamond (001) Surface by Rutherford Backscattering Spectroscopy. Jpn. J. Appl. Phys..

