# Supplementary material for: CH3NH3PbBr3 Perovskite Single-Crystal X-Ray Photon-Counting Detection Based on Multi-Layer Electrodes
Source: Sensors (Basel). 2026 May 11;26(10):3030. doi: 10.3390/s26103030 (PMC13210985; doi:10.3390/s26103030)
Supplement: Supplementary file 1 [file sensors-26-03030-s001.zip › sensors-4260909-supplementary.pdf]

Supporting information for

$CH_3NH_3PbBr_3$ Perovskite Single-Crystal X-Ray Photon-Counting Detection Based on Multi-Layer Electrodes

Songchao Wang[1,2] Hanwen Zhang[3], Gangyi Chen[2], Yuzhu Pan[5], Yulian Zhang[5], Qianqian Huang[6], Jinbao Chen[1,4] and Xin Wang[1,4*]

1 National Key Laboratory of Aerospace Mechanism, Nanjing University of Aeronautics and Astronautics, Nanjing 210016, China.
2 Shanghai Institute of Spacecraft Equipment, Shanghai, 200000, China
3 Shanghai Aerospace Equipments Manufacturer Co.Ltd, Shanghai 200245, China
4 College of Physics, Nanjing University of Aeronautics and Astronautics, Nanjing 210016, China.
5 Key Laboratory of Semiconductor Display Materials and Chips, Suzhou Institute of Nano-Tech and Nano-Bionics, Chinese Academy of Sciences, Suzhou 215123, China
6 School of Information Technology, Jiangsu Open University, Nanjing 210017, China
* Correspondence: xin-wang@nuaa.edu.cn;

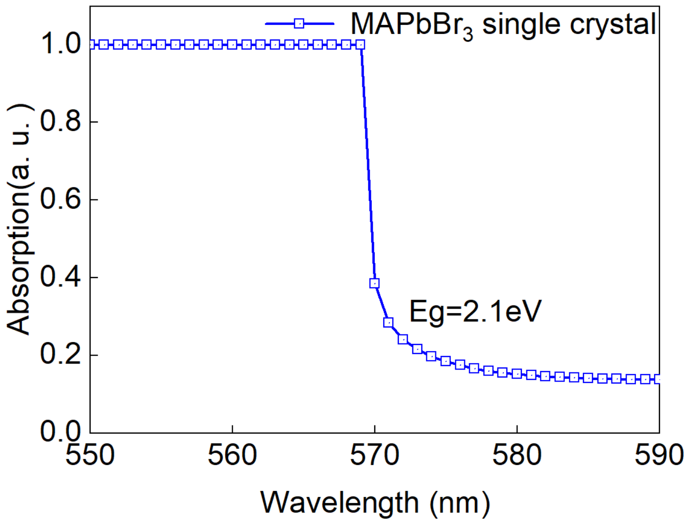

Figure S1. Absorption curve of $MAPbBr_3$ single crystal.

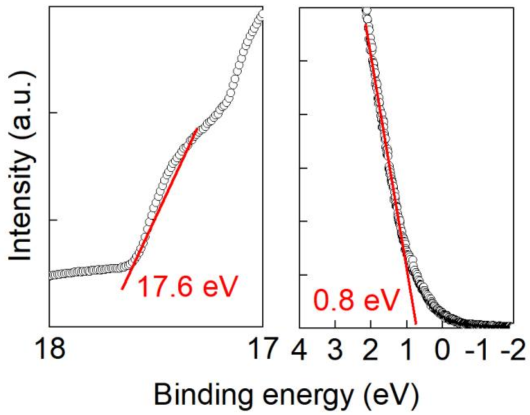

Figure S2. UPS result of $MAPbBr_3$ single crystal.

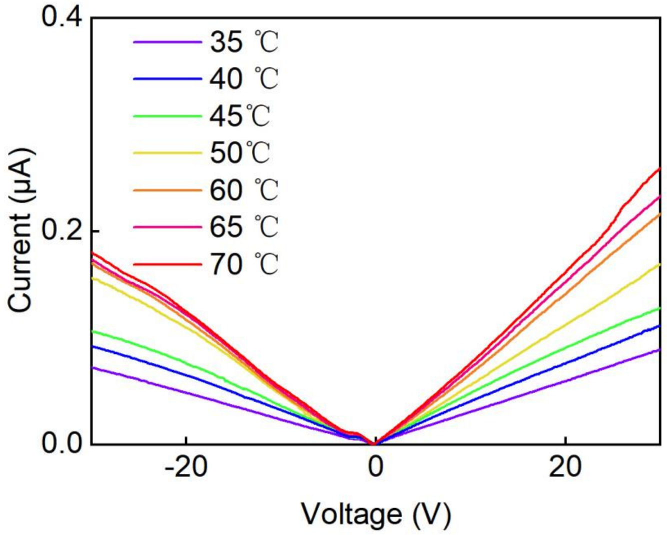

Figure S3. Temp-dependent I-V characterization of Au/Pt/Ti device.

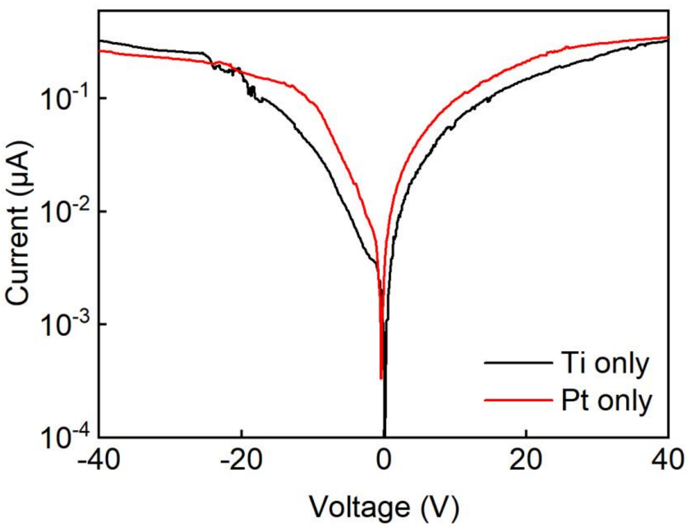

Figure S4: I-V characterization results of $MAPbBr_3$ single crystal with only Ti and Pt electrodes.

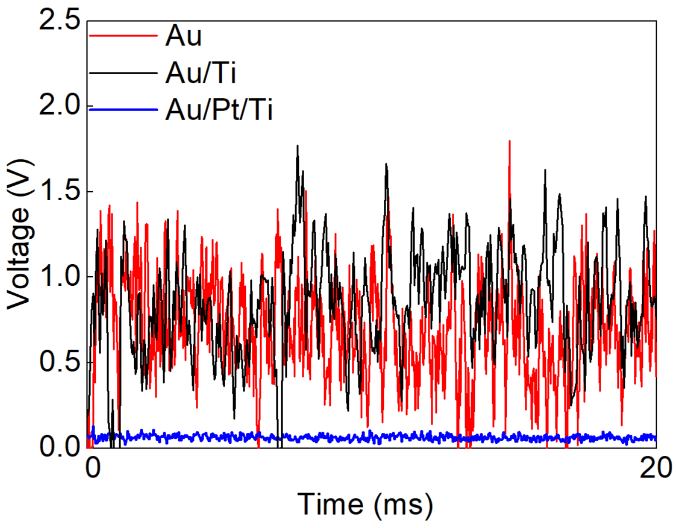

Figure S5. Noise of Au, Au/Ti and Au/Pt/Ti under -50 V bias.

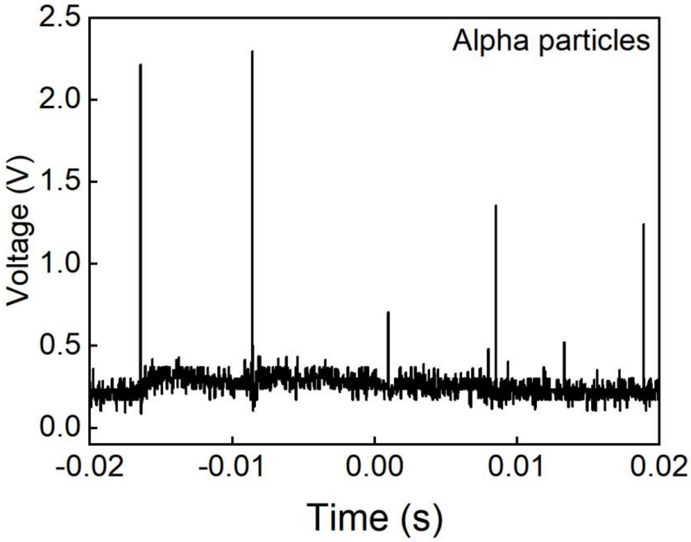

Figure S6. Alpha response of Au/Pt/Ti device.

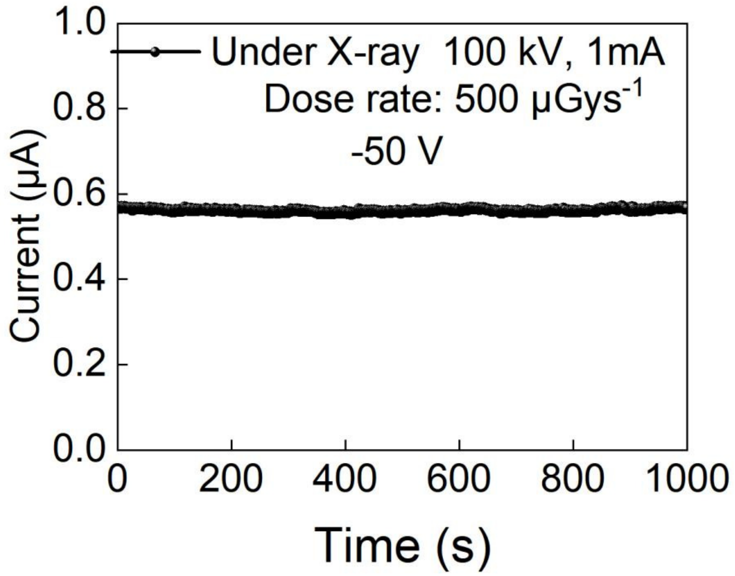

Figure S7: X-ray irradiation stability.
